# Wear resistance and surface roughness assessment of CAD/CAM graphene-reinforced PMMA versus conventional PMMA crowns

**DOI:** 10.1186/s12903-025-07125-5

**Published:** 2025-11-22

**Authors:** Marwa Beleidy, Ahmed Ziada

**Affiliations:** 1 Department of Fixed Prosthodontics, Faculty of Dentistry, October 6 University, Giza, Egypt; 2 Department of Fixed Prosthodontics, Faculty of Dentistry, Benisuef University, Benisuef, Egypt

**Keywords:** CAD/CAM PMMA, Graphene-reinforced PMMA, Wear, Surface roughness, Non-contact profilometry, 3D surface deviation

## Abstract

**Background:**

To evaluate the graphene-reinforced PMMA wear resistance and surface roughness compared to PMMA after accelerated artificial aging.

**Methods:**

Twenty molar-shaped crowns were fabricated (*n* = 10): graphene-reinforced PMMA (G) and PMMA (P), and an intraoral scanner digitized them. After thermomechanical aging, equivalent to 6 months of clinical service, the same scanner rescanned each crown. 3D analysis software calculated the wear loss volume of tested crowns. Twenty virtual discs were designed and milled from the two tested materials (*n* = 10). The discs were subjected to thermocycling, equivalent to 6 months of clinical service. Before and after thermocycling, a non-contact optical profilometer quantified the surface topography of all discs for assessment of surface roughness (Ra). Data were analyzed using ANOVA, Student’s t, and Bonferroni’s post-hoc tests (*P* ≤ 0.05).

**Results:**

Both 2D and 3D analysis approaches showed no statistically significant difference in surface wear between the P and G groups (*P*-value = 0.053, Effect size = 1.268; *P*-value = 0.649, Effect size = 0.271), respectively. Surface roughness did not differ statistically between the two materials independent of thermocycling (*P*-value = 0.172, Effect size = 0.150). Mean Ra decreased significantly after thermocycling in both groups regardless of material type (*P*-value = 0.035, Effect size = 0.320). For PMMA, the mean Ra did not change significantly after thermocycling. While mean Ra decreased significantly following the thermocycling of graphene-reinforced PMMA.

**Conclusions:**

Both conventional PMMA and graphene-reinforced PMMA exhibited comparable performance in terms of surface wear resistance and surface roughness, regardless of artificial aging.

## Background

Interim restorations serve essential purposes beyond their temporary functionality. They facilitate mastication and phonation, protect against mechanical and thermal injuries, and act as a barrier to microbial penetration, thereby maintaining pulpal and periodontal integrity. They preserve a patient’s appearance and confidence, particularly in the anterior regions. Furthermore, they facilitate the clinical evaluation of fit, function, and aesthetics prior to the production of the ultimate prosthesis. In dental procedures with prolonged treatment durations, such as full-mouth rehabilitation, interim materials must possess sufficient durability for sustained use. This emphasizes the necessity for enhanced surface integrity and material strength to guarantee optimal aesthetics, durability, and performance, considering the complex oral environment.

Polymethyl methacrylate (PMMA) has been extensively employed in prosthodontics as a provisional material owing to its low modulus of elasticity, less wear on opposing surfaces, aesthetic appeal, ease of repair, cost-effectiveness, and relatively fast production procedure [1]. Nonetheless, its mechanical constraints—especially in terms of wear resistance, surface durability, and susceptibility to bacterial infection—have necessitated the investigation of material improvements [2].

Innovations in computer-aided designing/computer-aided manufacturing (CAD/CAM) technology and material reinforcement through the incorporation of inorganic fillers have created new opportunities for PMMA performance [3]. Nanographene oxide (nGO) exhibits exceptional biocompatibility, remarkable mechanical strength, superior fracture toughness, and outstanding wear resistance that can substantially enhance the longevity of dental restorations when incorporated into PMMA matrices [4–6]. The incorporation of graphene into PMMA matrices is significantly dependent on the chemical interactions between the functional groups of graphene, particularly in nGO, and the polymer chains of PMMA. The oxygen-containing functional groups on graphene oxide, including hydroxyl and carboxyl groups, facilitate hydrogen bonding and van der Waals interactions with the ester groups of PMMA, thereby enhancing dispersion and interfacial adhesion while maintaining consistent mechanical performance and processability [7–11]. Moreover, graphene may alter the polymerization kinetics of PMMA by acting as a radical scavenger, thereby modifying the degree of conversion and cross-link density, which in turn impacts material stability [12, 13]. Nonetheless, obstacles persist, including graphene’s propensity to agglomerate at elevated concentrations, potential cytotoxicity, and a limited understanding of its long-term chemical stability, as well as its effects on polymerization shrinkage and surface roughness.

Wear, which denotes the material degradation of a surface, is a crucial factor in the performance of dental restorations. It constitutes a process involving multiple factors that can be categorized as either physiologic or pathologic concerning intraoral circumstances. An ideal restorative material must exhibit wear properties similar to those of natural teeth, as excessive wear can compromise both cosmetic quality and practical performance [14, 15]. The provisional restoration exhibits uneven occlusal contacts due to the wear process, resulting in dental over-eruption and a reduction in both occlusal clearance and vertical dimension of occlusion. Several parameters, including modulus of elasticity, fracture toughness, hardness, size, volume, and the hardness of the filler, influence the material’s wear characteristics [16].

In provisional restorations, surface roughness plays a critical role in facilitating plaque formation and bacterial colonization, especially around the margins of dental restorations. This ultimately leads to periodontal inflammation and infection. Alterations in this physical property significantly influence bacterial adhesion.

Despite the increasing interest in graphene-reinforced PMMA composites, a significant gap persists in the literature about their mechanical properties and clinical efficacy for dental prosthesis applications [17, 18]. The objective of this study was to assess the enhancement of PMMA when reinforced with nGO, specifically in terms of wear resistance and surface roughness, which are crucial properties for prosthodontic applications. The first research hypothesis posited that the graphene-reinforced PMMA would exhibit significantly improved wear resistance compared to conventional PMMA. The second reaseach hypothesis was that the graphene-reinforced PMMA would demonistrate reduced surface roughness compared to conventional PMMA.

## Methods

The protocol for this study was approved by the Research Ethics Committee of the Faculty of Dentistry, October 6th University (No. RECO6U/25–2023).

Two CAD/CAM materials were tested: PMMA (PMMA DISK; Yamahachi Dental MFG., Aichi Pref., Japan) and graphene-reinforced PMMA (G-CAM; Graphenano Dental, Valencia, Spain).

The power analysis used crown wear as the primary outcome for crown specimens. According to the findings of Güven ME et al., the mean and standard deviation (SD) for wear in the GR and PMMA groups were 0.9 (0.23) and 0.5 (0.2) mm^3^, respectively [19]. Utilizing an alpha (α) level of 5% and a beta (β) level of 0.8 (Power = 80%), the effect size (d) was 1.86. The minimum predicted sample size was 6 crown specimens for each group, which was increased to 10 specimens in both groups, permitting compensation for specimen loss during artificial aging.

In contrast, the power analysis used surface roughness (Ra) as the primary outcome measure for disc specimens. The findings from Tasın S et al. indicated that the mean and standard deviation (SD) for Ra in the PMMA and milled groups were 0.43 (0.07) and 0.32 (0.06) µm, respectively [20]. Utilizing an alpha (α) level of 5% and a beta (β) level of 0.8 (Power = 80%), the effect size (d) was 1.86. The minimum estimated sample size was 7 disc specimens for each group, which was increased to 10 specimens in both groups, allowing compensation for any specimen’s loss after artificial aging. All sample size calculations were conducted utilizing a sample size calculation software (G*Power; Version 3.1.9.2, HHUD, Germany).

For wear resistance analysis, a typodont mandibular first molar underwent preparation for an all-ceramic restoration [21]. Twenty dental epoxy resin dies were subsequently produced [22]. Each fabricated die underwent scanning with a three-dimensional (3D) dental scanner (Identica Hybrid T500; MEDIT Corp., Seoul, Korea). Twenty crowns were designed (exocad Dental CAD v3.0; exocad GmbH, Darmstadt, Germany) and then milled using a 5-axis milling machine (K5; vhf camfacture AG, Ammerbuch, Germany), 10 milled from PMMA blanks (PMMA DISK; Yamahachi Dental MFG., Aichi Pref., Japan) and 10 milled from graphene-reinforced PMMA blanks (G-CAM; Graphenano Dental, Valencia, Spain). The cement spacing was established at 50 μm. After milling, all crowns underwent abrasion with 50 μm Al_2_O_3_ particles at a 10 mm distance and 45º angle for 20 s (basic Quattro IS; Renfert, Hilzingen, Germany) [23]. They were later immersed in an ultrasonic bath (L&R Transistor/Ultrasonic T-14; L&R Ultrasonics, NY, USA) for 5 min. The crowns, fitted to corresponding dies, were assessed using optical magnification loupes.

The refinement and polishing were performed on the exterior surfaces of crowns using a disc-shaped silicone polisher (Universal Polisher 9627.900.220; Komet, SC, USA). They were then thoroughly polished using the technician’s handpiece, a goat hairbrush, and a polishing paste (Acrypol; Bredent, Senden, Germany), followed by achieving high-gloss polishing using a wool brush and a polishing paste (Abraso-Starglanz; Bredent, Senden, Germany) [24]. Then, the crowns underwent ultrasonic cleaning in distilled water (L&R Transistor/Ultrasonic T-14; L&R Ultrasonics, NY, USA) for 5 min, followed by desiccation with oil-free compressed air. They were later allowed to air dry at room temperature for a minimum of 4 weeks [25].

The dies and crowns were prepared for cementation by sandblasting (CEMAT NT4; Wassermann Dental Maschinen GmbH, Hamburg, Germany) [25]. The crowns were subsequently positioned and cemented to their dies using self-adhesive resin luting cement (G-CEM; GC, IL, USA) with a loading device, applying a 250 g weight for 1 min [22].

For surface roughness assessment, a virtual disc (10 mm diameter × 25 mm thickness) was created using computer software (exocad DentalCAD 3.1 Rijeka; Exocad GmbH, Darmstadt, Germany) and subsequently converted into a stereolithography file. After the milling process (DWX-52D Plus; Roland DGA Corp., CA, USA), CAD/CAM polymer cylinders were sliced into sections using a cutter (Secotom 10 Isomet; Struers, Ballerup, Denmark) to produce standard discs (10 mm diameter × 2 mm thickness) for each group. The finishing and polishing of discs were executed as previously stated [24].

The crowns underwent assessment for two-body wear utilizing a dual-axis chewing simulator (four-station multimodal ROBOTA chewing simulator; ROBOTA INDUSTRIES, Alexandria, Egypt) [19]. The simulator incorporated a thermo-cyclic protocol employing a servomotor (model ach-09075dc-t; AdTech Technology Co., Shenzhen, China). Six mm diameter steatite ceramic spheres (Steatite grinding balls; CeramTec, Plochingen, Germany) were utilized as antagonists [26]. The specimens were installed and subjected to sequential testing under a 5 kg weight, equivalent to 49 N of chewing force, for 120,000 cycles, mimicking 6 months of clinical service according to the wear testing parameters [26].

Furthermore, the discs were subjected to accelerated artificial aging in a thermocycling apparatus (Thermocycler TC4; SD Mechatronik GmbH, Feldkirchen-Westerham, Germany) for 5,000 cycles in distilled water at 5 °C and 55 °C for 30 s, with a 10-sec transfer time between two baths, simulating 6 months of clinical service [27].

**Fig. 1:**
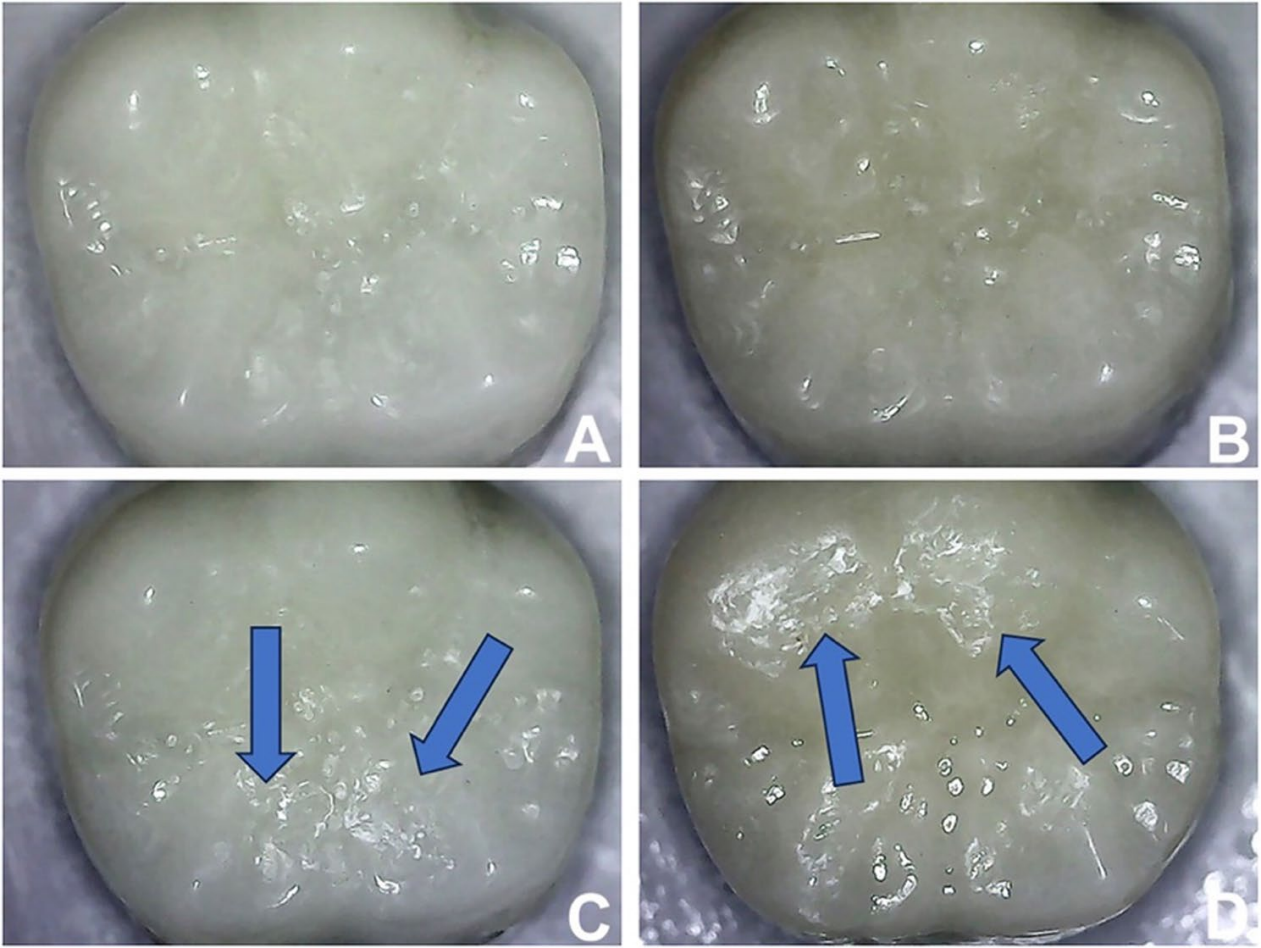
PMMA and graphene-reinforced PMMA crowns before (**A** and **B**) and after thermomechanical aging (**C** and **D**), respectively. Blue arrows indicate the area of occlusal wear after aging

Following thermomechanical aging, the crowns underwent a visual inspection for any failures using a stereomicroscope (Eclipse E600; Nikon Corp., Tokyo, Japan) (Fig. 1C and D). Subsequently, the specimens were subjected to ultrasonic cleaning in distilled water for 3 min. Through the same operator and using the same tactic, the crowns’ surfaces were scanned both before and after the wear test. Then, the volumetric loss (mm^3^) was calculated. Also, the discs’ surfaces were evaluated quantitively for surface roughness before and after thermocycling aging.

**Fig. 2:**
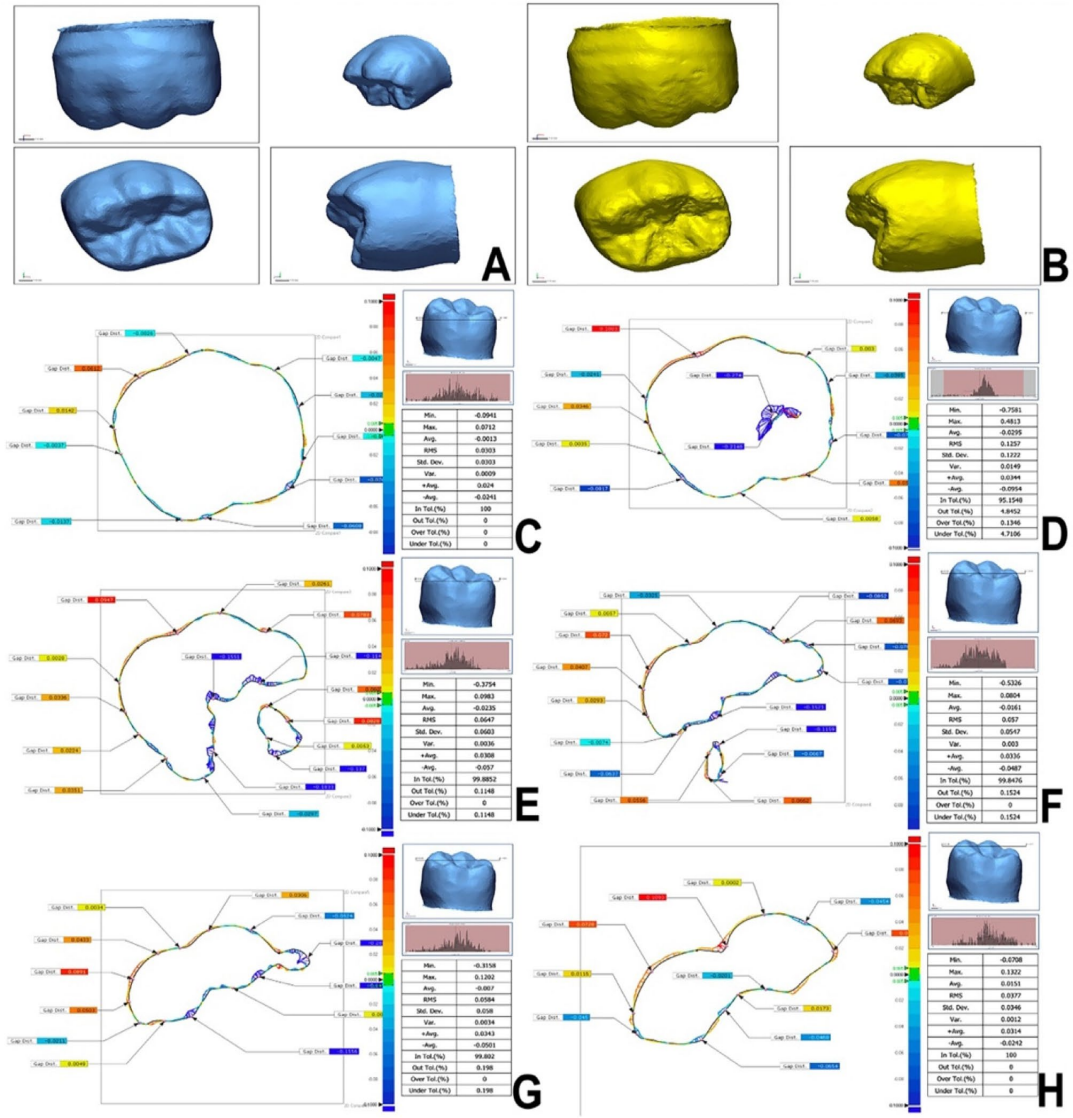
CAD Reference Model/reference (**A**) and CAD test Model/measured (**B**) datasets of tested specimen. Six cross-sections (**C**-**H**) for 2D comparison of superimposed reference and measured datasets showing the geometric dimensional changes. The color difference map displays a ± 100 μm range (20 color segments) and a ± 5 μm tolerance range (green). The location of the cross-section concerning the crown is shown in the upper right corner

For 3D surface deviation wear analysis, a powder (Renfert Scanspray; Renfert GmbH Co., Hilzingen, Germany) was applied to the specimens for profiling purposes. A 3D dental scanner (Identica Hybrid T500; MEDIT Corp., Seoul, Korea) with a Mono 2.0 MP resolution and < 7 μm accuracy scanned all specimens to produce the 3D standard tessellation language (STL) models [26]. Following their import, the digital 3D models underwent analysis (Geomagic Design X 2024.3.1; 3D Systems, SC, USA). The optimum 3D mesh models produced from 3D scanning data were transformed into CAD surface parts. (Fig. 2A and B) [26].

Changes in geometric dimensions and wear measurements were assessed through a 3D comparative analysis of superimposed datasets, comparing the CAD reference model (CRM) with the CAD test model (CTM/measured) before and after the wear test, focusing on the x-, y-, and z-coordinates. The initial alignment for each specimen was established between both datasets using Enhanced Alignment Accuracy with Feature Recognition to ensure that standard 3D reference matches and computerized fit were present. A 2D comparison was performed, analyzing 6 cross-sections with tolerance values set at 5 μm (Fig. 2C-H). This approach facilitated the extraction of numerical values to determine the average and maximum depth values, as well as to calculate the volume loss (mm³).

All data point clouds were used to calculate the root mean square (RMS) that represents the 3D difference between CRM and CTM utilizing the following equation [26]:

$$RMS\;=\;\frac1{\sqrt n}\;\times\;\sqrt{\sum\limits_{i=1}^n\left({\text{X}}_{1,i}\;-\;{\text{X}}_{2,i}\;\right)^2}$$

In this context, X1, i represents the measurement point i of the CRM, X2, i denotes the measurement point i of the CTM, and n indicates the total number of measurement points analyzed in each evaluation.

The RMS value represented the observed divergence from zero between two distinct sets [26]. The low RMS data demonstrated effective 3D superimposition. A color difference map featuring a range of ± 100 μm (20 color segments) and a tolerance range of ± 5 μm (green), which signifies no change, was employed to depict each 3D comparison (Fig. 2). The red area indicating positive deviation (5 μm to 100 μm) suggests that the CTM measurements surpassed the CRM data, emphasizing gain areas. The blue area, which signifies negative deviations (− 5 μm to − 100 μm), indicates that the CTM data were lower than the CRM data, highlighting areas of loss. The green area (± 5 μm) delineated the exact areas that underwent precise scanning. The horizontal displacement was calculated as the root mean square (RMS) within the theoretical plane.

**Fig. 3:**
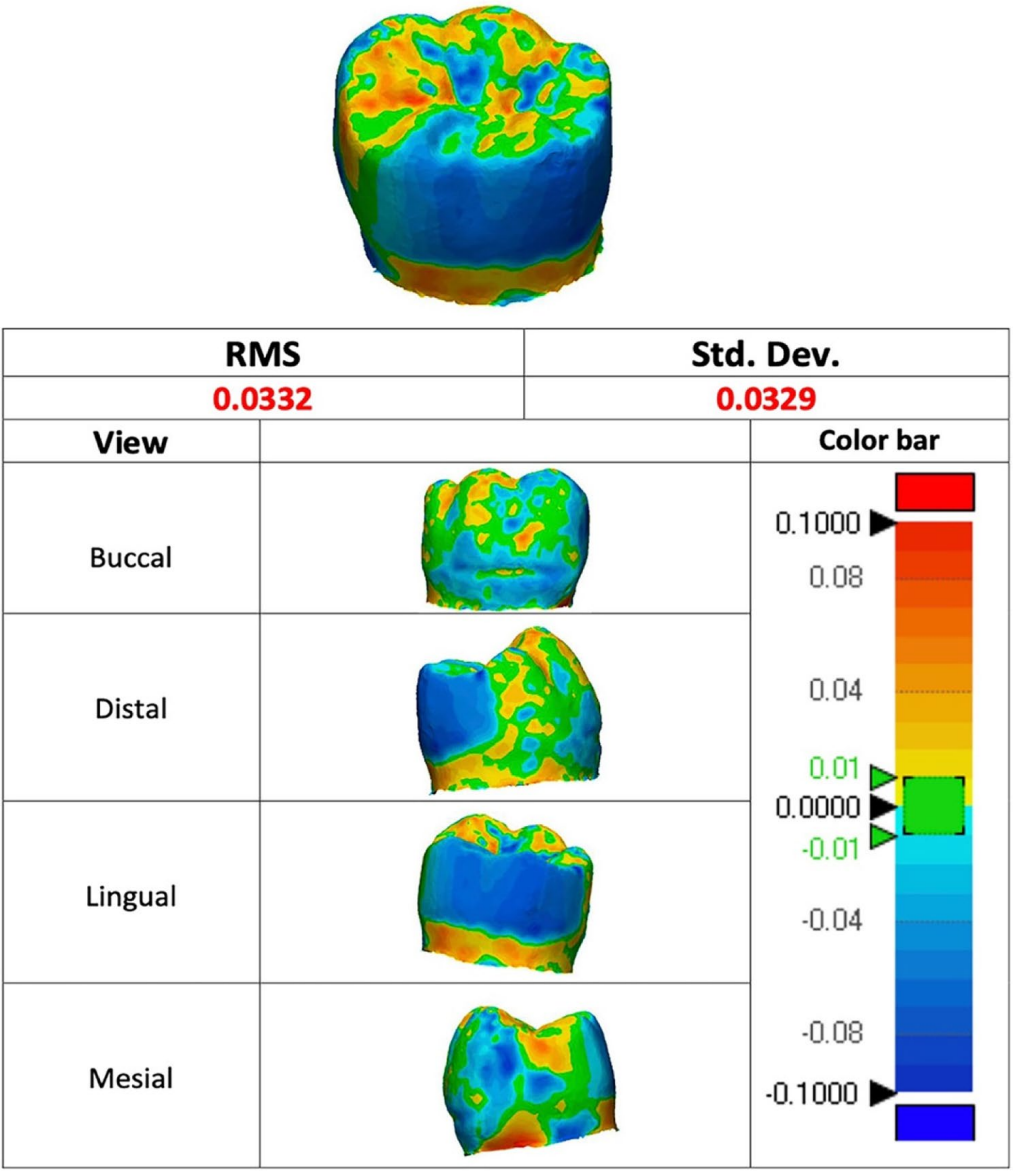
Color maps generated after superimpositions in 3D. Overcontoured areas are indicated in red color, and under-contoured areas are shown in blue color. Acceptable areas with a ± 10 μm tolerance range are marked in green

The software’s “3D Compare Tool” was utilized for the quantitative 3D evaluation of deviations on the exterior surfaces, generating color maps (Fig. 3). Regions that are over-contoured are indicated in red. In contrast, those that are under-contoured are shown in blue, with deviation thresholds set at a maximum and minimum of 100 μm. The tolerance range has been set at 10 μm and is indicated in green.

**Fig. 4:**
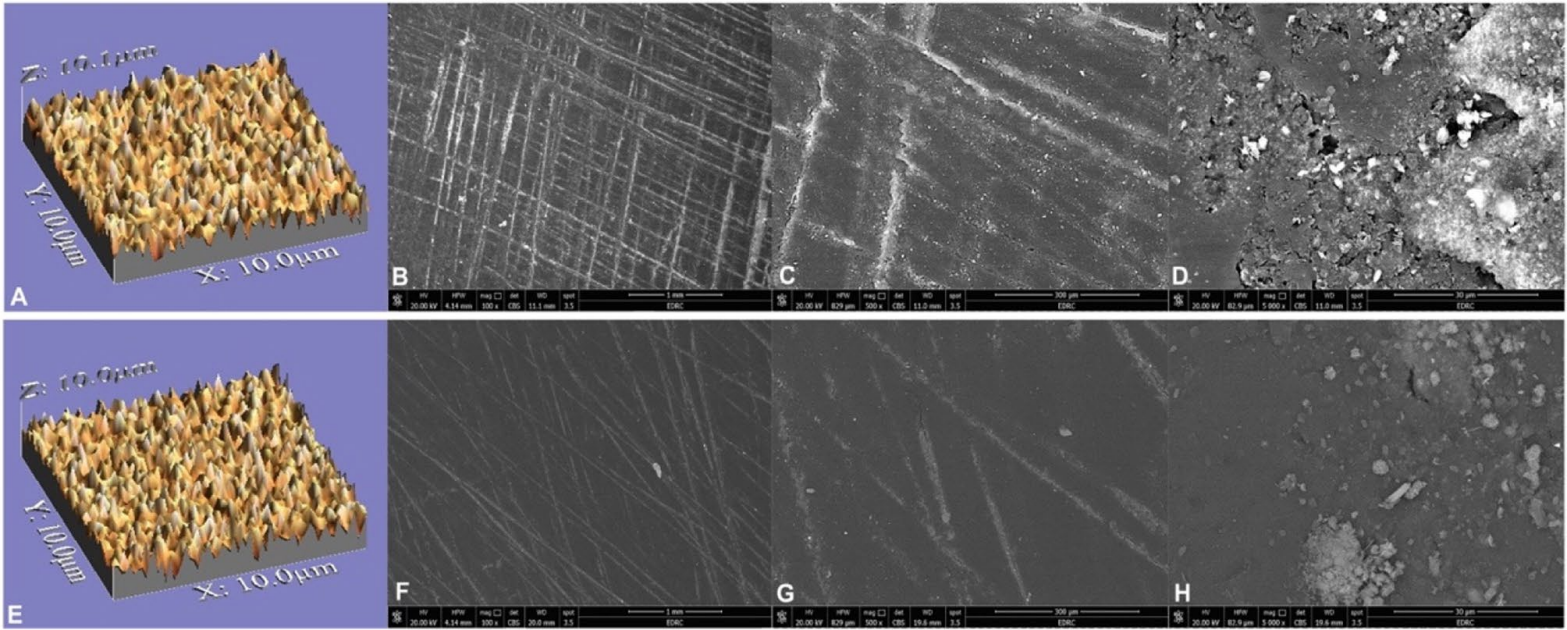
3D surface topographic and scanning electron microscope (100×, 500×, and 5000× magnifications, respectively) images of tested PMMA discs before (**A**-**D**) and after thermocycling (**E**-**H**). Polished specimens before thermocycling presented rougher surfaces. Some scratches left by abrasives were observed (**B**, **C**, **F**, and **G**)

**Fig. 5:**
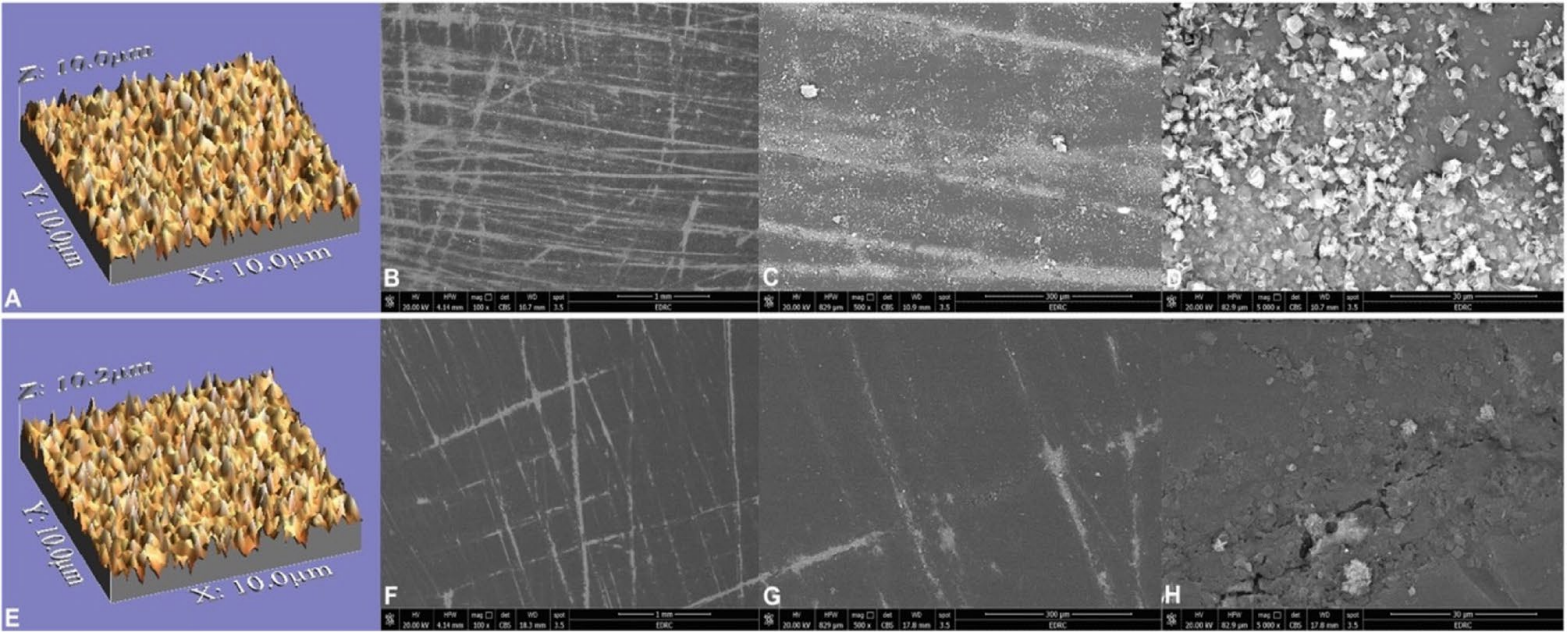
3D surface topographic and scanning electron microscope (100×, 500×, and 5000× magnifications, respectively) images of tested graphene-reinforced PMMA discs before (**A**-**D**) and after thermocycling (**E**-**H**). Polished specimens before thermocycling presented rougher surfaces. Some scratches left by abrasives were observed (**B**, **C**, **F**, and **G**)

For surface roughness analysis, a non-contact optical approach was used to measure the surface topography of all discs. A 90× USB digital microscope (U500X USB 2.0, 500x magnification; OEM, Guangdong, China) equipped with a 3 MP camera and connected to a suitable computer captured 20 images for each specimen [27, 28]. The camera was set vertically at a height of 2.5 cm and positioned at a 90° angle relative to each specimen. Eight adjustable LED bulbs were employed to achieve a nearly 95% color rendering index. The shots were captured at 1280 × 1024 pixels and cropped to 350 × 400 pixels. A data processing software (WSxM Ver 5 develop 4.1; Nanotec) was used to evaluate cropped photos in 3D, creating 10 × 10 μm images that resemble bacterial adherence to restoration surfaces in vivo [29]. The calibration process transformed pixels into µm to estimate average heights (Ra), a valid indicator of surface roughness (Figs. 4 and 5, A and E).

For scanning electron microscopy (SEM) imaging, two polished discs from each group, before and following thermocycling aging, were arbitrarily selected for imaging. A scanning electron microscope (JSM-6610 LV; JEOL, Tokyo, Japan) was used to analyze the specimens, which were coated with gold via sputtering (Quorum Q150R; Quorum Technologies, East Sussex, England). Images were acquired at 20 kV with magnifications of 100×, 500×, and 5000× (Figs. 4 and 5, B, C, D, F and G, and H) [29].

The distribution of numerical data was assessed for normality using the Kolmogorov-Smirnov and Shapiro-Wilk tests. The data demonstrated a normal (parametric) distribution. The data were presented as mean values with standard deviations (SD). The Student’s t-test was utilized to analyze the differences in wear between the two types of materials. The ANOVA test was used to analyze the effects of material type, thermocycling, and their interactions on the mean (Ra). Bonferroni’s post-hoc test was used for pairwise comparisons following a significant ANOVA test. The significance threshold was set at *P* ≤ 0.05. A statistical analysis was performed utilizing statistical software (IBM SPSS Statistics 23.0; IBM Corp., NY, USA).

## Results

The analysis of wear resistance of the tested crowns (mm³) revealed no statistical significance in wear between the two groups, regardless of whether 2D or 3D methods were employed (*P*-value = 0.053, Effect size = 1.268; *P*-value = 0.649, Effect size = 0.271) (Table 1).

**Table 1:** Descriptive statistics of student’s t-test for comparison between wear resistance (2D and 3D) results between PMMA (P) and G-PMMA (G) groups

Method	Group (*P*)		Group (G)		*P*-value	Effect size (d)
	Mean	SD	Mean	SD		
2D	0.0605	0.0022	0.0682	0.0082	0.053	1.268
3D	0.0330	0.0042	0.0341	0.0038	0.649	0.271

* Significant at *P* ≤ 0.05

Repeated measures ANOVA for surface roughness (µm) of tested discs indicated that material type, regardless of thermocycling, did not have a statistically significant effect on mean Ra (*P* = 0.172, Effect size = 0.150). Thermocycling significantly affected mean Ra (*P* = 0.035, Effect size = 0.320) across all material types. The interaction of variables did not significantly affect the mean Ra (P-value = 0.309, Effect size = 0.086). The lack of statistical significance in the interaction between the variables indicates their independence (Table 2).

**Table 2:** The mean, standard deviation (SD) values of repeated measures ANOVA test for the main effects of the two variables (material types and thermocycling) on surface roughness (Ra) (µm)

Variables		Mean (µm)	SD	Type III Sum of Squares	df	Mean Square	F-value	*P*-value	Effect size (Partial Eta squared)
Material type	Group (P)	0.2513	0.0200	0.001	1	0.001	2.112	0.172	0.150
	Group (G)	0.2604	0.0251						
Thermocycling	Before	0.2661	0.0184	0.003	1	0.003	5.647	0.035*	0.320
	After	0.2455	0.0222						
Material type x Thermocycling interaction				0.001	1	0.001	1.129	0.309	0.086

*df*degrees of freedom = (*n*-1)

*Significant at *P* ≤ 0.05

The analysis of the impact of various variable interactions on Ra, both before and after thermocycling, revealed no statistically significant difference between the two material types. Regarding Group (P), the analysis showed no statistically significant alteration in mean Ra following thermocycling (*P*-value = 0.371, Effect size = 0.067). In Group (G), a statistically significant reduction in mean Ra was observed following thermocycling (*P*-value = 0.032, Effect size = 0.330) (Table 3).

**Table 3:** The mean, standard deviation (SD) values of repeated measures ANOVA test for comparison between surface roughness (Ra) with different interactions of variables (before and after thermocycling)

Thermocycling	Group (*P*)		Group (G)		*P*-value	Effect size (Partial eta squared)
	Mean	SD	Mean	SD		
Before thermocycling	0.2570	0.0152	0.2753	0.0175	0.058	0.2670
After thermocycling	0.2455	0.0231	0.2454	0.0231	0.993	0.0001
*P*-value	0.371		0.032*			
Effect size *(Partial eta squared)*	0.067		0.330			

* Significant at *P* ≤ 0.05

## Discussion

### Methodology

An optimal restorative material should exhibit wear characteristics comparable to natural dentition [15]. The absence of hardness and toughness in PMMA polymer resins results in inadequate wear resistance, compromising the durability of the components. Additionally, a significant limitation for their long-term use is associated with inadequate antimicrobial properties, which may lead to polymer deterioration and potential infections upon contact with tissues or during implantation for restoration [2].

The integration of nanotechnologies in dentistry has garnered significant attention. Nanographene-reinforced PMMA consists of graphene, a crystalline form of carbon that creates a densely packed lattice with a large surface area resembling a honeycomb structure [30]. This distinctive structure improves the mechanical properties of PMMA [30].

For the tested crowns, polishing aimed to achieve a stable exterior surface and enhance the wear resistance of tested materials through improved surface roughness. In another study, none of the specimens were polished, aiming to assess the inherent wear resistance of examined materials following artificial aging [19]. Moreover, standardizing and polishing complex geometries, such as crowns, poses significant challenges [25].

Utilizing human enamel cusps as antagonistic abraders for wear testing may lack standardized robustness [31]. Consequently, steatite balls were used due to their demonstrated efficacy as antagonists in the literature [32]. To address the incorrect replication of steatite balls to the complex enamel structure, their hardness, akin to that of enamel, was chosen (steatite: 680 HV, enamel: 330 HV) [32].

The incorporation of both 2D and 3D wear analysis provided a comprehensive assessment of the tested material’s wear. 2D analysis, often profilometric, yielded linear measurements of wear depth or cross-sectional area, facilitating the quantification of localized surface alterations. Conversely, 3D analysis quantified volumetric wear and topographical changes throughout the entire surface, providing a more clinically relevant representation of total material loss during functioning and aligning with digital workflows commonly used in restorative dentistry. Emphasizing 3D analysis may be more suitable for mimicking intraoral conditions, although 2D analysis remains significant for accurate comparison evaluations in controlled experimental settings. Both collectively augment the robustness of wear characterization by integrating localized precision with extensive spatial information [26, 33].

### Wear resistance

The first research hypothesis was rejected, as no statistically significant difference between PMMA and G-PMMA in either 2D (0.0605 ± 0.0022 mm³ vs. 0.0682 ± 0.0082 mm³) or 3D (0.033 ± 0.0042 mm³ vs. 0.0341 ± 0.0038 mm³) measurements was observed when considering volume loss at the worn area.

Considering the different chemical compositions of the tested materials, and although G-CAM’s composition is not disclosed, the presence of graphene was expected to influence wear resistance compared to conventional PMMA. Graphene has demonstrated the ability to improve mechanical properties such as tensile strength, flexural modulus, and hardness in various polymer matrices. However, its effectiveness in PMMA seems to be highly dependent on concentration, dispersion quality, and interfacial bonding [34–36].

Graphene serves as an effective reinforcement in composites, significantly enhancing mechanical characteristics at a weight concentration of 2.5% [37]. Conversely, concentrations of graphene in PMMA exceeding 0.35% resulted in high discoloration, which is unsuitable for optimal dental aesthetics [38]. This would pose a clinical trade-off between reinforcement and aesthetics in graphene-reinforced PMMA materials for dental or prosthetic applications. Similar to the use of graphene-reinforced PMMA, a previous study analyzed the concentration of graphene through Raman spectra and quantified it as 0.027% by weight [39]. This concentration showed no improvement in the mechanical properties of graphene-reinforced PMMA compared to conventional PMMA in the current investigation. Therefore, the current concentration likely reflects a balance aimed at preserving crown aesthetics rather than maximizing mechanical performance.

Even at low concentrations, inadequate dispersion can lead to nanoparticle agglomeration. These clusters may act as stress concentrators rather than reinforcing agents, thereby diminishing the potential benefits of graphene incorporation [40]. Moreover, PMMA does not inherently form strong chemical bonds with graphene, unless functionalization techniques are employed [41, 42]. Such weak interfacial bonding impairs effective load transfer from the polymer matrix to the graphene filler, particularly under wear conditions.

Moreover, wear resistance is a complex, multifactorial property influenced by surface hardness, modulus, plasticity, and interfacial fatigue resistance [43]. If the addition of graphene improves one parameter but compromises another (e.g., embrittlement or altered polymerization kinetics), the overall wear behavior may remain unchanged or even worsen.

In another study, researchers found greater volume loss of graphene-reinforced PMMA compared to pure PMMA [20]. The variations in wear test parameters might result in differences detected in the present study. Additionally, the scanner’s inherent inaccuracy might have a potential influence on the results as a different scanner was used [20]. In this investigation, the analysis did not assess the maximum wear depth; thus, the higher nonsignificant volume loss of the G group does not necessarily indicate a deeper worn area, as volume loss may have occurred on a broader surface, considering the circular motion of the chewing simulator. Worn areas were evident at various locations on the occlusal surfaces of the crowns, despite attempts to standardize the indenter’s contact point location during aging (Fig. 1), lending credence to this result. Such heterogeneity mirrors clinical situations where masticatory dynamics and individual occlusal anatomy contribute to variable wear patterns, reinforcing the clinical relevance of the observed results.

### Surface roughness

Surface roughness is clinically significant, as it influences microbial plaque retention, thereby increasing the risk of surface fatigue and reducing biocompatibility. Alterations in surface roughness potentially mitigate the risks of caries, gingival, periodontal, and peri-implant diseases [44]. The second research hypothesis was rejected. The mean Ra values for graphene-reinforced PMMA (0.2604 ± 0.0251 μm) and conventional PMMA (0.2513 ± 0.02 μm) were statistically comparable pre- and post-thermocycling, suggesting that the nanoscale incorporation of graphene oxide did not yield a clinically meaningful change in surface texture. This might be due to the homogenization method, nanoparticle distribution, and effective polishing significantly influencing surface roughness levels. In alignment with the present study’s findings, Ionescu et al. and others have found that pre-polymerized PMMA and graphene-reinforced PMMA exhibited comparable Ra values, irrespective of aging effects [44, 45]. In contrast, other investigations have shown that graphene increased the resin’s surface roughness, which might arise from graphene’s difficulty in dispersing within a polymer matrix, potentially leading to agglomerates with elevated graphene concentrations [46, 47].

Furthermore, the lack of significant change in surface roughness post-thermocycling is clinically relevant, as thermocycling is designed to simulate intraoral thermal stresses and aging over time. The slight reduction in Ra observed in the graphene-reinforced group following thermocycling may be attributed to graphene’s effect in enhancing microhardness resulting from the incorporation of graphene nanoparticles into the matrix. However, differences in surface roughness and hardness are contingent upon the fabrication method and surface treatments employed [48]. Moreover, the thermocycling did not degrade the material surface roughness, this implies structural integrity of the matrix through homogeneous nanoparticles dispersion.

Still, the assessed materials demonstrate resistance to extended exposure, with 5000 cycles corresponding to 6 months of intraoral service. From a clinical perspective, the mean Ra levels for each group are slightly above 0.2 μm, which is the critical threshold above which plaque accumulation increases significantly [49]. However, some studies have reported that surface roughness above this value does not necessarily correlate with higher plaque levels [50, 51]. Other factors such as surface free energy, hydrophobicity, and chemical composition also modulate bacterial adhesion and biofilm development [52, 53]. Therefore, surface roughness should be considered as one of multiple interacting factors influencing oral biofilm dynamics.

### Limitations

The in vitro study demonstrated notable limitations, as it failed to mimic the intricate nature of the intraoral environment, which encompasses the evaluation of the impact of materials on surface energy, biofilm formation, antimicrobial activity, and cellular behavior. The test design focused on two-body wear; however, an alternative strategy, including three-body wear, such as brushing, may influence the results. Additionally, a single type of antagonist was used for thermomechanical aging, which may have influenced wear. This study did not analyze the antagonists. The volume loss appraisal depended on a single experienced operator for all digital analyses, which may have influenced the results. Additionally, different scanner resolutions and polishing protocols may reveal a notable impact. Further clinical investigations are needed to validate the interpretations drawn from the results of this study assessing the long-term clinical outcomes to elucidate graphene’s role in dental prosthetic materials.

## Conclusions

Both conventional PMMA and graphene-reinforced PMMA exhibited comparable performance in terms of surface wear resistance and surface roughness, regardless of artificial aging.

## Data Availability

The datasets used and/or analyzed during the current study are available from the corresponding author upon reasonable request.
